# The Disorganization of Retinal Inner Layers Is Correlated to Müller Cells Impairment in Diabetic Macular Edema: An Imaging and Omics Study

**DOI:** 10.3390/ijms24119607

**Published:** 2023-06-01

**Authors:** Edoardo Midena, Tommaso Torresin, Stefano Schiavon, Luca Danieli, Chiara Polo, Elisabetta Pilotto, Giulia Midena, Luisa Frizziero

**Affiliations:** 1 Department of Ophthalmology, University of Padova, 35128 Padova, Italy; tommaso.torresin@gmail.com (T.T.); stefano.schiavon.6@studenti.unipd.it (S.S.); chiara.polo@studenti.unipd.it (C.P.); elisabetta.pilotto@unipd.it (E.P.); lfrizziero@gmail.com (L.F.); 2 IRCCS—Fondazione Bietti, 00198 Rome, Italy; luca.danieli@fondazionebietti.it (L.D.); giulia.midena@fondazionebietti.it (G.M.)

**Keywords:** retina, imaging, inflammation, disorganization of retinal inner layers, macula, proteomics, aqueous humor, diabetic macular edema, Müller cell, GFAP

## Abstract

The disorganization of retinal inner layers (DRIL) is an optical coherence tomography (OCT) biomarker strictly associated with visual outcomes in patients with diabetic macular edema (DME) whose pathophysiology is still unclear. The aim of this study was to characterize in vivo, using retinal imaging and liquid biopsy, DRIL in eyes with DME. This was an observational cross-sectional study. Patients affected by center-involved DME were enrolled. All patients underwent spectral domain optical coherence tomography (SD-OCT) and proteomic analysis of aqueous humor (AH). The presence of DRIL at OCT was analyzed by two masked retinal experts. Fifty-seven biochemical biomarkers were analyzed from AH samples. Nineteen eyes of nineteen DME patients were enrolled. DRIL was present in 10 patients (52.63%). No statistically significant difference was found between DME eyes with and without DRIL, considering the AH concentration of all the analyzed biomarkers except for glial fibrillary acidic protein (GFAP), a biomarker of Müller cells dysfunction (*p* = 0.02). In conclusion, DRIL, in DME eyes, seems to strictly depend on a major dysfunction of Müller cells, explaining its role not only as imaging biomarker, but also as visual function Müller cells-related parameter.

## 1. Introduction

The International Diabetes Federation estimates that the total number of adults living with diabetes worldwide is currently around 537 million and will reach around 643 million by 2030. Diabetic retinopathy (DR) and diabetic macular edema (DME) are the most common ophthalmic complications of diabetes mellitus (DM) [1].

The pathophysiology of DME is multifactorial and, despite great progress, it has not yet been fully elucidated. For a long time, DME has been considered a microvascular complication of DM, a hypothesis supported by the fact that ophthalmoscopic signs secondary to DM preferentially affect the retinal vascular tree [2]. However, other factors significantly—and probably earlier—influence the progression of diabetic retinopathy and maculopathy, including retinal neuroinflammation with the activation of resident cells, in particular Müller and microglial cells, which anticipate the vascular clinical manifestations of DR and the clinical appearance of DME [3].

The recognition of fine retinal imaging biomarkers useful in qualifying a specific pathogenetic mechanism or cell type involvement, and eventually capable of predicting the risk of development and progression of DME, is important for screening, monitoring, and treating these eyes. Optical coherence tomography (OCT) can provide both quantitative and qualitative assessments of various retinal parameters, including the disorganization of retinal inner layers (DRIL) [4]. DRIL has been proposed as a biomarker strongly associated with visual acuity in patients with DME. However, unfortunately, its pathophysiology is still under debate, with contrasting hypotheses [5,6]. Although the importance of both OCT and biochemical biomarkers for managing and treating DME is known in the literature, still, few studies correlate qualitative and quantitative OCT biomarkers with biochemical ones, obtained by sampling aqueous humor (AH), in patients with DME [7,8,9].

The aim of this study was to contribute to improving our understanding of the pathophysiology of DRIL, analyzing the expression of several AH biomarkers in patients affected by DME, with and without DRIL.

## 2. Results

Population

This study included 19 eyes of 19 patients (12 male and 7 female subjects). The main characteristics of the study population are summarized in Table 1.

**Figure 1:**
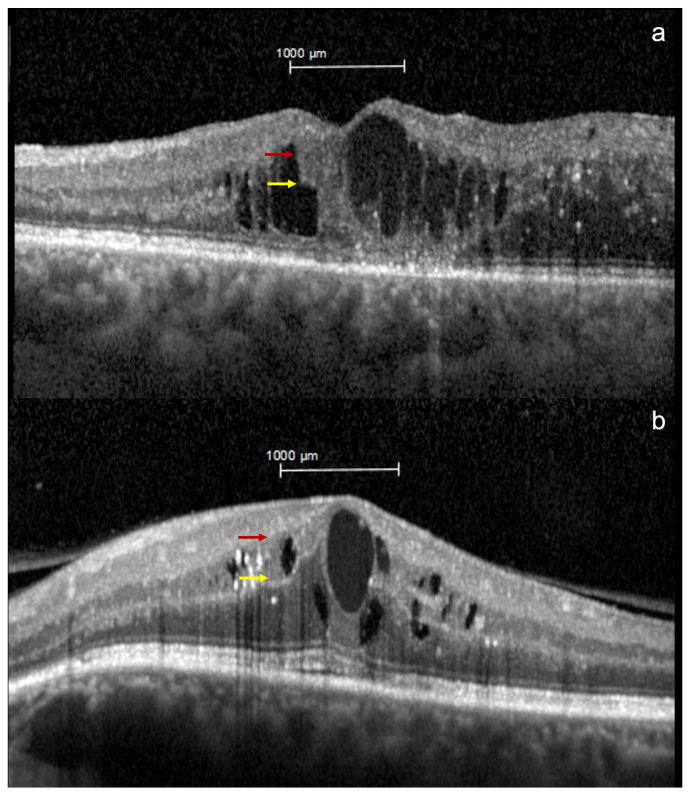
Detection of disorganization of retinal inner layers (DRIL) on optical coherence tomography (OCT). OCT scans obtained using Spectralis HRA + OCT (Heidelberg Engineering, Heidelberg, Germany) of (**a**) a patient with DME with DRIL and (**b**) a patient with DME without DRIL. Red and yellow arrows show the boundary between the ganglion cell—inner plexiform layer complex and inner nuclear layer (INL), and between the INL and outer plexiform layer, respectively, within the central 1000 μm. The boundaries are (**a**) not distinguishable in the first scan and (**b**) completely distinguishable in the second one.

Mean CST was 496.89 ± 151.47 µm and mean MV was 0.39 ± 0.12 µm^3^. The mean visual acuity was 70.37 ± 8.23 ETDRS score. DRIL was present in 10 patients (52.63%) and absent in 9 (47.37%) (Figure 1). No statistically significant difference was found between DME eyes with and without DRIL considering the main clinical characteristics analyzed (Table 1).

With regards to the biochemical biomarkers analyzed in the AH, a statistically significant difference was found in glial fibrillary acid protein (GFAP) values between DME eyes with and without DRIL (*p* = 0.02) (Table 2). No other statistically significant difference was found between the two groups, considering all other biomarkers analyzed in the AH (Table 2).

## 3. Discussion

DME is the leading cause of legal blindness in diabetic patients and can occur at any stage of DR. The use of SD-OCT in clinical practice represents the gold standard non-invasive imaging modality in the evaluation and management of DME [10]. Some SD-OCT features have been identified and may serve as biomarkers for diagnosing and predicting morphologic and visual outcomes in patients with DME [11]. One of the major limitations of SD-OCT alone is to provide an image without any possibility of understanding the biologic changes occurring in the retinal microenvironment of DME eyes.

The awareness of the multiple mechanisms involved in the pathogenesis and progression of DME has been recently confirmed by the increase in clinical studies aimed at identifying the mediators of these mechanisms in the ocular fluids of diabetic subjects [9]. AH samples can provide information with minimal risk as an outpatient procedure, allowing the identification of biomarkers correlated with intraretinal processes and the related morphologic features [7,9].

DRIL was first described as an OCT sign by Soliman et al., and proposed as a surrogate marker correlating with current visual acuity in individuals with existing or resolved center-involved DME [12]. It was later defined as “the horizontal extent in microns for which any boundaries between the GCL-IPL, INL, and OPL could not be identified”. The same authors reported reproducibility of DRIL extent grading among three independent masked graders ranging from 0.80 to 0.86 [5].

Jolitkov et al. found that DRIL was associated with reduced visual acuity, contrast sensitivity, and standard automated perimetry performance, even without DME, conferring to DRIL the status of a reliable and readily available biomarker for monitoring neuro-retinal impairment in DME [13].

Besides the diagnostic significance of DRIL, it has also been proposed as a prognostic biomarker of anatomical and functional outcomes [14]. Das et al. found that the presence of DRIL correlated with a worse visual outcome for each 100 µm increase in the lateral extension of DRIL [15]. Moreover, different patterns of DRIL resolution have been shown to differently correlate with VA changes after edema resolution [16,17]. Sun et al. found that the resolution of DRIL after 4 months of treatment with intravitreal anti-VEGF was predictive of better visual outcomes in DME after 12 months [5].

DRIL was hypothesized to represent the disruption of cells within inner retinal layers, indicating the interruption of pathways that transmit visual information from the photoreceptors to the ganglion cells [5]. Disruption has been hypothesized to result when bipolar cells exceed their elasticity limit due to edema [6]. In this study, we did not find a significant difference in markers of neurons survival and growth (e.g., neurotrophins) and cell death regulation (TNF, Fas Ligand) in patients with and without DRIL. Indeed, all patients were naïve (previously untreated) with a first time detection of center-involved macular edema, probably without any long term damage involving neurons, even in presence of DRIL. Moreover, we did not find any difference in markers of extracellular matrix regulation (e.g., TIMP, TACE), tissue remodeling (e.g., osteopontin), and vasculo- and angiogenesis (VEGF, PDGF), suggesting that these mechanisms are not differently activated in patients with or without DRIL. Finally, markers of microglia activation (IL-1, IL-6, TNFα), leucocytes migration (e.g., ICAM), leucocytes chemotaxis (e.g., RANTES, MIP, MCP), and molecules recognition (TLR) were also not differently expressed in the two groups of DME patients (with and without DRIL) [18,19].

Conversely, we found a significant difference in GFAP expression, a protein produced by Müller cells activated during DME [20,21]. This intermediate filament protein is mostly expressed in the normal retina by astrocytes. In diabetes, it has been well-established that Müller cells become activated early on and one of the most prominent signs of Müller cell activation is the (increased) expression of GFAP, whereas astrocytes precociously undergo cell death by apoptosis [22,23]. Müller cells’ nuclei are located in the inner nuclear layer but their processes involve the whole retinal thickness and are in contact with different retinal elements. They produce factors capable of modulating blood flow, vascular permeability, cell survival, and neuroprotection, besides extra and intracellular water transport. Therefore, injury to these cells has an essential role in the pathogenesis of early microcirculatory abnormalities and neurodegeneration, as shown in DME [24]. GFAP values obtained from AH samples are significantly increased in human eyes with diabetes, confirming that Müller cells are precociously correlated with decreased GFAP values detected in the AH, demonstrating that DME resolution may contribute to restore Müller cells function through changes in Müller cell’s metabolic activity [25,26,27,28].

From both a morphologic and functional point of view, each Müller cell constitutes the backbone and the functional regulator of a column of retinal neurons, representing the smallest functional unit of visual signaling [29]. Moreover, they act as living optical fibers guiding light through the inner retinal layers towards photoreceptors [29]. Recently, a correlation between DRIL length and the severity of metamorphopsia, evaluated with M-CHARTS, confirmed the relevance of the inner retinal layers in visual function [30]. These units are progressively damaged during DME development with rapid alterations in the size and structure of synapses and cell bodies of both neurons and Müller cells [29]. The loss of the retinal physiological microstructure may be revealed as disorganization of the retinal layers and the reduced visualization of their boundaries at SD-OCT. The population of this study was a selected group of naïve DME patients, with recent visual symptoms and center involvement. In this type of recent, active, clinically significant DME, DRIL seems to characterize eyes with a more significant morphological disruption of Müller cell, rather than other retinal components, such as microglia or neuronal cells. In fact, the study of multifocal electroretinogram in DME patients has shown a correlation between DRIL and reduced P1 amplitude, which depends on Müller cells [31]. Moreover, Müller cells appear to be more elastic than neurons, and the restoration of their morphology following treatment could be imaged at SD-OCT as “restoring” DRIL [16,29]. This seems to confirm the possible origin of DRIL from Müller cells, rather than simply bipolar cells changes, at least until DRIL shows recovering potential. Then, neuron cells function and integrity may also be involved, without any restoration possibility. Studies investigating DRIL after a dexamethasone implant for DME found a significant improvement in DRIL after treatment, possibly related to a positive architectural effect on Müller cells [14,32].

One of the main limitations of this study is the sample size. Indeed, this is a pilot study, providing, for the first time, a biologic proof of concept of a morphological feature with increasing clinical relevance. The study of AH biomarkers requires a procedure for both the sampling and the subsequent analysis, which is safe and well standardized but in any event more costly and time-consuming compared to fully non-invasive diagnostic imaging, and thus limiting the numerosity of the study group [9]. However, among the 57 AH biomarkers analyzed only GFAP obtained a statistically significance even in a small population, underlining the relevance of this factor in DME eyes affected by DRIL. Further studies are needed to confirm the results of this pilot study.

## 4. Materials and Methods

### 4.1. Population

This was an observational cross-sectional study with prospective enrollment. Patients affected by type 2 diabetes were enrolled from September 2019 to June 2021. Informed consent was obtained from all patients and the study respected the principles of the Declaration of Helsinki. The study was approved by the Ethics Committee of the Azienda Ospedale Università di Padova (Prot. 3194/AO/14).

Inclusion criteria were: ≥18 years old male and/or female subjects diagnosed with type 2 DM, based on the diagnostic criteria established in 2011 by the WHO (World Health Organization) [33]; presence of untreated center-involved DME; and adequate quality of the retinal images.

Exclusion criteria were: visual symptoms prior to 3 months, proliferative DR, vision-limiting ocular conditions other than DR (such as amblyopia, age-related macular degeneration, myopic degeneration, retinal dystrophies, optic neuropathies, retinal vascular diseases, advanced glaucoma, or corneal opacity of any cause); previous laser treatment; previous intravitreal injection therapy (any drug); history of previous ocular trauma or surgery except for uncomplicated cataract extraction (>12 months earlier); significant media opacity, precluding adequate evaluation of the fundus and instrumental imaging; high refractive defect (>6.0 D); poorly controlled systemic arterial hypertension; systemic neurodegenerative diseases (multiple sclerosis, Alzheimer’s disease, etc.).

### 4.2. Imaging

All patients underwent a full ophthalmologic evaluation, including spectral domain-OCT (SD-OCT), using Spectralis (Spectralis HRA + OCT; Heidelberg Engineering, Heidelberg, Germany). An OCT macular map scan-pattern was used, with a 20° × 20° scan area centered onto the fovea. Central subfield thickness was automatically calculated by the device in the central 1 mm diameter circular zone centered onto the fovea (CST). The macular volume (MV) was automatically obtained as the overall 6 × 6 volume.

DRIL was defined as an alteration in the normal architecture of the internal retinal layers in which a clear distinction among the ganglion cell–inner plexiform layer complex (GCL-IPL), inner nuclear layer (INL), and outer plexiform layer (OPL) is not recognized in the millimeter centered onto the fovea, as previously defined [5,6]. The evaluation was dichotomous: presence/absence of DRIL. The presence/absence of DRIL was analyzed by two independent masked operators; in case of disagreement, the case was discussed with a third operator until agreement was reached.

### 4.3. Omics Data Acquisition and Analysis

All patients underwent paracentesis of the anterior chamber and AH sampling, in an operating room setting, following a standardized preoperative procedure: disinfection of the periocular skin with iodopovidone 10% (ESO-JOD; ECOLAB, Agrate Brianza, Italy), irrigation of the conjunctival sac with iodopovidone 5% for two minutes (Oftasteril, Alpha Intes, Italy), and washing with balanced saline solution (BSS; Alcon, Fort Worth, TX, USA). A small amount of AH (150–200 µL) was withdrawn from the anterior chamber using an insulin needle (30 Gauge). The AH samples were placed inside a micro vial containing 10 µL of a cocktail of protease inhibitors (Pierce Biotechnology, Rockford, IL, USA), labeled to be able to assign each sample to the correct patient, and rapidly stored at −80 °C until analysis. The protein content was evaluated using a digital spectrophotometer (NanoDrop; Thermo Fisher Scientific Inc., Waltham, MA, USA) and the protein concentration was calculated using a linearized standard curve (BSA) and the A280 software (version 3.8.1). Subsequently, the samples underwent sonication (VibraCell; Sonica, Newton, CT, USA) and centrifugation to be able to collect the clear supernatant (13,000 rpm/7 min). The subsequent specific biomarkers analyzed were chosen after revision of the existing literature to identify biomarkers that could be representative of each category even considering practical and economic reasons.

### 4.4. Glass-Chip Protein Array Analysis

The biochemical biomarker analysis used a customized, glass-chip protein array (RayBiotech^TM^ technology) supplied by the manufacturer (Norcross, GA, USA). The normalized and prediluted AH samples were loaded onto the array according to the manufacturer’s instructions: the slides were allowed to incubate overnight at approximately 4 °C, washed and exposed to a biotinylated antibody mixture, followed by the addition of a solution containing cy3-streptavidin; all these steps were performed above a special orbital motion shaker (Certomat II; Sartorius AG, Göttingen, Germany) with the hybridization/washing solutions provided by the kit. The slides were washed with MilliQ water, centrifuged, and scanned with a special microarray device (Molecular Devices LLC, Sunnyvale, Silicon Valley, CA, USA). To obtain an adequate Cy3/Cy5 ratio (specific signal/background signal), all slides were scanned referring to previously validated parameters and procedures. The fluorescence signals were acquired using the special GenePix 4100 microarray scanner (Molecular Devices, LLC, Sunnyvale, Silicon Valley, CA, USA) equipped with the GENEPIX pro 3.0 software (Axon Instrument, Foster City, CA, USA). From the fluorescence values obtained, the “tails” were excluded from the evaluation, including only the median value as a reference value for each biomarker change. An intra- and inter-assay coefficient of variability <10% was established, so that at each signal increase of 1.5 times, or a reduction of the same of 0.65 times, were considered to guarantee an adequate signal above the background one. The fluorescence signals were then analyzed. Fifty-five biomarkers were quantified including inflammatory interleukins (IL-1β, 4, 6, 8, 10, 11, 12p40, 12p70, 13, 17A, 17B, 17C, 17E), Tumor Necrosis Factor (TNF)α, TNFβ, Interferon (INF) γ, Tissue Inhibitor of Metalloproteinase (TIMP)-1, TIMP-2, TIMP-4, TNFα converting enzyme (TACE), Neural cell adhesion molecule 1 (NCAM-1), Intracellular Adhesion Molecule (ICAM)-2, ICAM-3, Vascular cell adhesion protein (VCAM)-1, Osteopontin, Insulin, Insulin regulated and normal T cell Expressed and Secreted (RANTES), Macrophage Inflammatory Protein (MIP)-1α, MIP-1β, MIP-1δ, MIP-3α, MIP-3β, Toll-Like Receptor (TLR)-2, Monocyte Chemo-attractant Protein (MCP)—1, Glial cell-Derived Neurotrophic Factor (GDNF), Brain-Derived Neurotrophic Factor (BDNF), NeuroTrophin (NT)-3, NT-4, Granulocyte-Colony Stimulating Factor (G-CSF), Macrophage-CSF (M-CSF), VEGF A, VEGF C, VEGF D, Tumor growth factor (TGF) β1, Platelet-Derived Growth Factor (PDGF)-BB, Epidermal Growth Factor (EGF), soluble Tumor Necrosis Factor Receptor (sTNF R)1, sTNF R2, VEGF Receptor (VEGF-R)1, VEGF-R2, Insulin-like Growth Factor (IGF)—1, Fas Ligand (Fas L), Erythropoietin (EPO), and Pigment epithelium-derived factor (PEDF).

### 4.5. Immunoprecipitation and SDS PAGE Analysis

According to the direct immunoprecipitation (IP) technique, the capture antibodies of interest, inwardly rectifying potassium channel (Kir) 4.1 and GFAP, were incubated with Pure Proteome Protein G Magnetic beads (15 µL; Millipore, Burlington, MA, USA) and immobilized with a magnet, to generate the antibody–beads complex. The beads-bound antibodies were then added to the normalized samples (30 µg total protein) and after 2 h incubation, the captured proteins were washed and eluted in denaturing Loading Buffer. All steps were performed under orbital shaking (Certomat II; Sartorius AG). Loading Buffer and samples were preheated at 90 °C for 10 min and loaded on 4–12% precasted SDS-PAGE gels (Bio-Rad Laboratories Inc., Hercules, CA, USA) and electrophoresis was performed in a MiniProtean3 apparatus (Bio-Rad Laboratories Inc., Hercules, CA, USA) under reducing conditions (120 V/frontline). Electrophoresed bands were transferred to 0.22 µm membranes (Hybond; GE Healthcare, Buckinghamshire, UK) at 12 V/40 min in a semidry Trans-Blotting apparatus (Bio-Rad Laboratories Inc., Hercules, CA, USA). Membranes were stained with the highly sensitive Sypro Ruby protein blot stain (Invitrogen, Waltham, MA, USA), according to a standard procedure, to visualize and acquire the specific bands. Immunoblotting followed by chemiluminescent detection was additionally performed to better visualize the protein of interest. In both cases, the optical density (OD) was checked using the freely available ImageJ software (Image J v1.43; NIH http://rsb.info.nih.gov/ij/, accessed on 1 October 2021). Data were saved as 8-bit TIFF files and exported for figure assembly using the Adobe Photoshop 2022 22.0.0 software release (Adobe Systems Inc., San Jose, CA, USA).

### 4.6. Statistical Analysis

Patient characteristics and study parameters were summarized according to the usual methods provided by descriptive statistics: mean and standard deviation for quantitative variables, absolute and relative (percentage) frequency for the qualitative ones. The comparisons between DME patients with and without DRIL were made by means of Wilcoxon–Mann–Whitney test for independent samples. Tests were interpreted as statistically significant if *p* < 0.05 or a different value where specified. SAS^®^ 9.4 software (SAS Institute, Cary, NC, USA) was used for all statistical analyzes.

## 5. Conclusions

In conclusion, using AH sampling as a liquid biopsy approach, we provided insights into the pathophysiology of an imaging biomarker, DRIL, which has recently gained growing clinical relevance. The possibility of assessing Müller cells’ modifications in DME and their response to treatments, directly with OCT, may be of great relevance, making DRIL a biomarker not only of prognostic significance but also for therapy timing, monitoring, and modulation.

## Figures and Tables

**Table 1:** Clinical characteristics of the study population.

		All Patients	DRIL	No DRIL	*p*-Value
**Age (years), mean ± SD**		68 ± 6.8	69.1 ± 6.9	66.8 ± 6.8	0.5387
**Sex**	male, *n* (%)	12 (63.2%)			
	female, *n* (%)	7 (36.8%)			
**Duration of DM (years), mean ± SD**		18.28 ± 8.96	18.6 ± 10.3	17.4 ± 7.3	0.8701
**Therapy**	oral hypoglycemics, *n* (%)	6 (31.58%)	2 (33.33%)	4 (66.67%)	0.6135 *
	insulin, *n* (%)	8 (42.10%)	5 (62.5%)	3 (37.50%)	
	oral hypoglycemics + insulin, *n* (%)	5 (26.32%)	3 (60%)	2 (40%)	
**HbA1c, mean ± SD**		7.2 ± 0.5	7.3 ± 0.3	7.1 ± 0.7	0.5945
**Lens status**	phakia, *n* (%)	9 (47.37%)			
	pseudophakia, *n* (%)	10 (52.63%)			
**Grading DR**	mild, *n* (%)	0 (0%)			
	moderate, *n* (%)	18 (94.73%)			
	severe, *n* (%)	1 (5.23%)			
**Eye**	right, *n* (%)	12 (63.16%)			
	left, *n* (%)	7 (36.84%)			

SD = standard deviation; *n* = numerosity; DM = diabetes mellitus; DR = diabetic retinopathy; * Fisher’s exact test

**Table 2:** Relationship between DRIL and biochemical biomarkers.

	DRIL		
Cytokine, Mean (SD)	Absent	Present	*p*-Value
Albumin	220.8 (59.0)	163.6 (151.8)	0.4379
BDNF	550.9 (612.1)	361.8 (242.9)	0.3913
EGF	255.9 (65)	250.4 (92.3)	0.8383
EPO	193.4 (55.4)	192.0 (63.7)	0.8383
Fas L	92.4 (16.5)	68.8 (30.4)	0.0538
G-CSF	781.3 (276.5)	810.7 (332.5)	0.7751
GDNF	349.8 (99.9)	370.7 (133.7)	0.7133
GFAP	129.8 (10.0)	151.8 (19.9)	0.0200
ICAM-2	1047.1 (629.6)	1366.2 (999.3)	0.6534
ICAM-3	370.5 (274.1)	500.1 (342.7)	0.5956
IGF-1	465.6 (60.2)	470.0 (135.2)	0.5956
IL-1β	46.3 (24.4)	47.3 (26.2)	0.9349
IL-4	90.2 (22.7)	86.0 (31.8)	0.6534
IL-6	1190.6 (180.2)	1368.1 (247.3)	0.0942
IL-8	1068.7 (293.9)	1559.1 (916.6)	0.4379
IL10	389.2 (44.6)	364.4 (120.2)	0.7133
IL11	390.3 (108.7)	353.5 (99.6)	0.4379
IL12p40	99.5 (31.9)	117.4 (42.6)	0.3689
IL12p70	166.9 (45.1)	206.6 (77.1)	0.2057
IL-13	473.0 (51.7)	496.7 (89.3)	0.4877
IL-17A	154.7 (35.3)	147.0 (58.4)	0.3913
IL-17B	655.2 (154.7)	621.9 (221.1)	0.6241
IL-17C	337.9 (102.6)	383.1 (123.8)	0.3913
IL-17E	317.6 (352.4)	264.1 (81.6)	0.2528
INF γ	1011.7 (347.1)	1098.1 (273.0)	0.3477
Insulin	738.4 (179.9)	797.9 (222.7)	0.3913
Kir 4.1	69.7 (16.1)	71.4 (18.2)	0.5956
MCP-1	11,292.1 (7189.9)	18,300.7 (11,946.6)	0.1779
MCSF	45.1 (25.3)	33.7 (15.3)	0.3913
MIP-1α	97.3 (32.6)	83.7 (38.8)	0.5675
MIP-1β	927.5 (564.0)	1350.6 (739.7)	0.0792
MIP-1δ	543.8 (653.3)	617.8 (896.4)	0.5403
MIP-3α	183.4 (41.3)	171.4 (83.2)	0.1779
MIP-3β	263.7 (253.0)	131.3 (139.0)	0.0942
NCAM-1	12,497.2 (7063.2)	12,646.6 (12,845.7)	0.3913
NT-3	175.9 (34.5)	187.6 (57.8)	0.7133
NT-4	383.8 (51.1)	341.6 (106.8)	0.2057
Osteopontin	15,553.9 (11,851.1)	11,047.8 (6862.2)	0.5956
PDGF-BB	84.5 (28.9)	96.0 (26.2)	0.1904
PEDF	18,871.6 (13,978.2)	14,161.7 (20,444.8)	0.4379
RANTES	109.4 (16.2)	128.0 (45.1)	0.6532
sTNF R1	273.2 (270.2)	294.8 (277.6)	0.6830
sTNF R2	738.0 (306.5)	799.6 (552.3)	0.9674
TACE	71.5 (15.7)	74.4 (23.5)	1.0000
TGFβ1	413.0 (55.8)	412.0 (117.3)	0.8383
TIMP-1	14,101.9 (2393.4)	15,996.4 (4910.3)	0.5956
TIMP-2	3477.8 (2827.7)	2913.6 (3397.9)	0.4877
TIMP-4	243.8 (83.1)	335.1 (190.8)	0.5956
TNFα	1157.7 (211.3)	1351.2 (331.6)	0.2057
TNFβ	1014.6 (213.0)	1167.6 (294.0)	0.3477
TLR-2	127.4 (26.1)	133.0 (31.4)	1.0000
VCAM-1	104.1 (94.4)	49.6 (72.4)	0.1739
VEGF-A	519.9 (288.0)	1253.1 (1150.9)	0.1309
VEGF-C	280.2 (279.0)	207.8 (93.9)	0.6241
VEGF-D	140.1 (46.1)	141.1 (57.8)	0.9674
VEGF-R1	162.9 (60.3)	290.0 (354.7)	0.9674
VEGF-R2	686.9 (247.5)	787.2 (593.1)	0.6534

SD: standard deviation; BDNF: Brain-Derived Neurotrophic Factor; EGF: Epidermal Growth Factor; EPO: Erythropoietin; Fas L: Fas Ligand; G-CSF: Granulocyte-Colony-Stimulating Factor; GDNF: Glial cell-Derived Neurotrophic Factor; GFAP: Glial Fibrillary Acid Protein; ICAM: Intracellular Adhesion Molecule; IGF: Insulin-like Growth Factor; IL: Interleukin; INF: Interferon; Kir: Inwardly rectifying potassium channel; MCP: Monocyte Chemo-attractant Protein; M-CSF: Macrophage-CSF; MIP: Macrophage Inflammatory Protein; NCAM: Neural cell adhesion molecule; NT: neurotrophin; PDGF: Platelet-Derived Growth Factor; PEDF: Pigment epithelium-derived factor; RANTES: Regulated And Normal T-cell Expressed and Secreted; sTNFR: soluble Tumor Necrosis Factor Receptor; TACE: TNFα converting enzyme; TGF: tumor growth factor; TIMP: Tissue Inhibitor of Metalloproteinase; TNF: Tumor Necrosis Factor; TLR: Toll-Like Receptor; VCAM: Vascular cell adhesion protein; VEGF: Vascular Endothelial Growth Factor; VEGFR: Vascular Endothelial Growth Factor Receptor. Significant results (level of significance 0.05) in bold.

## Data Availability

The data presented in this study are reported in the manuscript; any other reasonable request will be addressed by the corresponding author.

## References

[1] International Diabetes Federation (2019). IDF Diabetes Atlas.

[2] Das A., McGuire P.G., Rangasamy S. (2015). Diabetic Macular Edema: Pathophysiology and Novel Therapeutic Targets. Ophthalmology.

[3] Midena E., Pilotto E. (2017). Emerging Insights into Pathogenesis. Dev. Ophthalmol..

[4] Kwan C.C., Fawzi A.A. (2019). Imaging and Biomarkers in Diabetic Macular Edema and Diabetic Retinopathy. Curr. Diab. Rep..

[5] Sun J.K., Lin M.M., Lammer J., Prager S., Sarangi R., Silva P.S., Aiello L.P. (2014). Disorganization of the retinal inner layers as a predictor of visual acuity in eyes with center-involved diabetic macular edema. JAMA Ophthalmol..

[6] Sun J.K., Radwan S.H., Soliman A.Z., Lammer J., Lin M.M., Prager S.G., Silva P.S., Aiello L.B. (2015). Neural Retinal Disorganization as a Robust Marker of Visual Acuity in Current and Resolved Diabetic Macular Edema. Diabetes.

[7] Noma H., Funatsu H., Mimura T. (2012). Vascular endothelial growth factor and interleukin-6 are correlated with serous retinal detachment in central retinal vein occlusion. Curr. Eye Res..

[8] Lee H., Jang H., Choi A.Y., Kim H.C., Chung H. (2018). Association Between Soluble CD14 in the Aqueous Humor and Hyperreflective Foci on Optical Coherence Tomography in Patients with Diabetic Macular Edema. Investig. Ophthalmol. Vis. Sci..

[9] Midena E., Frizziero L., Midena G., Pilotto E. (2021). Intraocular fluid biomarkers (liquid biopsy) in human diabetic retinopathy. Graefes Arch. Clin. Exp. Ophthalmol..

[10] Im J.H., Jin Y.-P., Chow R., Yan P. (2022). Prevalence of diabetic macular edema based on optical coherence tomography in people with diabetes: A systematic review and meta-analysis. Surv. Ophthalmol..

[11] Hui V.W.M., Szeto S.K.F., Tang F., Yang D.M., Chen H., Lai T.Y.M., Rong A., Zhang S.M., Zhao P.M., Ruamviboonsuk P. (2022). Optical Coherence Tomography Classification Systems for Diabetic Macular Edema and Their Associations with Visual Outcome and Treatment Responses—An Updated Review. Asia Pac. J. Ophthalmol..

[12] Soliman A.Z., Radwan S.H., Prager S.G., Kwak H., Silva P.S., Aiello L.P., Sun J.K. (2012). Spectral Domain Optical Coherence Tomography Parameters Associated with Visual Acuity in Patients with Resolved Center-Involved Diabetic Macular Edema. Investig. Ophthalmol. Vis. Sci..

[13] Joltikov K.A., Sesi C.A., de Castro V.M., Davila J.R., Anand R., Khan S.M., Farbman N., Jackson G.R., Johnson C.A., Gardner T.W. (2018). Disorganization of Retinal Inner Layers (DRIL) and Neuroretinal Dysfunction in Early Diabetic Retinopathy. Investig. Ophthalmol. Vis. Sci..

[14] Zur D., Iglicki M., Sala-Puigdollers A., Chhablani J., Lupidi M., Fraser-Bell S., Mendes T.S., Chaikitmongkol V., Cebeci Z., Dollberg D. (2020). Disorganization of retinal inner layers as a biomarker in patients with diabetic macular oedema treated with dexamethasone implant. Acta Ophthalmol..

[15] Das R., Spence G., Hogg R., Stevenson M., Chakravarthy U. (2018). Disorganization of Inner Retina and Outer Retinal Morphology in Diabetic Macular Edema. JAMA Ophthalmol..

[16] Radwan S.H., Soliman A.Z., Tokarev J., Zhang L., van Kuijk F.J., Koozekanani D.D. (2015). Association of Disorganization of Retinal Inner Layers with Vision After Resolution of Center-Involved Diabetic Macular Edema. JAMA Ophthalmol..

[17] Pelosini L., Hull C.C., Boyce J.F., McHugh D., Stanford M.R., Marshall J. (2011). Optical coherence tomography may be used to predict visual acuity in patients with macular edema. Investig. Ophthalmol. Vis. Sci..

[18] Smith J.A., Das A., Ray S.K., Banik N.L. (2012). Role of pro-inflammatory cytokines released from microglia in neurodegenerative diseases. Brain Res. Bull..

[19] Midena E., Micera A., Frizziero L., Pilotto E., Esposito G., Bini S. (2019). Sub-threshold micropulse laser treatment reduces inflammatory biomarkers in aqueous humour of diabetic patients with macular edema. Sci. Rep..

[20] Sarthy V. (2007). Focus on molecules: Glial fibrillary acidic protein (GFAP). Exp. Eye Res..

[21] Vujosevic S., Micera A., Bini S., Berton M., Esposito G., Midena E. (2015). Aqueous Humor Biomarkers of Müller Cell Activation in Diabetic Eyes. Investig. Ophthalmol. Vis. Sci..

[22] Gallina D., Zelinka C.P., Cebulla C.M., Fischer A.J. (2015). Activation of glucocorticoid receptors in Müller glia is protective to retinal neurons and suppresses microglial reactivity. Exp. Neurol..

[23] Coughlin B.A., Feenstra D.J., Mohr S. (2017). Müller cells and diabetic retinopathy. Vision Res..

[24] Sundstrom J.M., Hernández C., Weber S.R., Zhao Y., Dunklebarger M., Tiberti N., Laremore T., Simó-Servat O., Garcia-Ramirez M., Barber A.J. (2018). Proteomic Analysis of Early Diabetic Retinopathy Reveals Mediators of Neurodegenerative Brain Diseases. Investig. Ophthalmol. Vis. Sci..

[25] Zhao M., Zhao S., Tang M., Sun T., Zheng Z., Ma M. (2022). Aqueous Humor Biomarkers of Retinal Glial Cell Activation in Patients With or Without Age-Related Cataracts and With Different Stages of Diabetic Retinopathy. Investig. Ophthalmol. Vis. Sci..

[26] Frizziero L., Calciati A., Midena G., Torresin T., Parrozzani R., Pilotto E., Midena E. (2021). Subthreshold Micropulse Laser Modulates Retinal Neuroinflammatory Biomarkers in Diabetic Macular Edema. J. Clin. Med..

[27] Rizziero L., Calciati A., Torresin T., Midena G., Parrozzani R., Pilotto E., Midena E. (2021). Diabetic Macular Edema Treated with 577-nm Subthreshold Micropulse Laser: A Real-Life, Long-Term Study. J. Pers. Med..

[28] Midena E., Bini S., Martini F., Enrica C., Pilotto E., Micera A., Esposito G., Vujosevic S. (2020). Changes of aqueous humor müller cells’ biomarkers in human patients affected by diabetic macular edema after subthreshold micropulse laser treatment. Retina.

[29] Reichenbach A., Bringmann A. (2013). New functions of Müller cells. Glia.

[30] Nakano E., Ota T., Jingami Y., Nakata I., Hayashi H., Yamashiro K. (2019). Correlation between metamorphopsia and disorganization of the retinal inner layers in eyes with diabetic macular edema. Graefes Arch. Clin. Exp. Ophthalmol..

[31] Khojasteh H., Riazi-Esfahani H., Pour E.K., Faghihi H., Ghassemi F., Bazvand F., Mahmoudzadeh R., Salabati M., Mirghorbani M., Esfahani M.R. (2020). Multifocal electroretinogram in diabetic macular edema and its correlation with different optical coherence tomography features. Int. Ophthalmol..

[32] Luís M.E., Sampaio F., Costa J., Cabral D., Teixeira C., Ferreira J.T. (2021). Dril Influences Short-term Visual Outcome after Intravitreal Corticosteroid Injection for Refractory Diabetic Macular Edema. Curr. Eye Res..

[33] American Diabetes Association (2013). Diagnosis and classification of diabetes mellitus. Diabetes Care.

